# The iron paradox and particle size sensing: transcriptomic response of *Enterococcus faecalis* to non-nutritive minerals

**DOI:** 10.1128/aem.00954-26

**Published:** 2026-08-19

**Authors:** Luhan Li, Jianchao Zhang, Sumin Qu, Zhiying Zhou, Beibei Wang, Haitao Wu, Zilin Guo, Lin Shi, Bohao Yin, Xiangyu Zhu, Yuebo Wang, H. Henry Teng

**Affiliations:** 1 School of Earth System Science, Institute of Surface-Earth System Science, Tianjin University, Tianjin, China; Colorado School of Mines, Golden, Colorado, USA

**Keywords:** non-nutritive minerals, transcriptomics, *Enterococcus faecalis*, iron acquisition, oxidative stress, particle size

## Abstract

Minerals are known to influence microbial metabolism as nutrient sources or redox partners, yet whether chemically inert, non-nutritive minerals can regulate microbial physiology, and through what mechanisms, remains poorly understood. Here, we used transcriptomics to investigate the response of *Enterococcus faecalis* to Al_2_O_3_ and SiO_2_ particles spanning nanoscale to millimeter scale. Mineral exposure triggered extensive transcriptional reprogramming across hundreds of differentially expressed genes. Notably, a massive upregulation of iron acquisition genes (log_2_FC range from 3.8 to 4.2) concurrent with oxidative stress defenses (catalase, thiol peroxidase, and NADH oxidase) is suggestive of an “iron paradox,” which is potentially attributable to mineral-mediated nutrient sequestration and steric hindrance of membrane transporters alongside interfacial reactive oxygen species generation. To adapt, *E. faecalis* orchestrated a coordinated metabolic shift, repressing serine catabolism (log_2_FC = −2.7) while investing nitrogen into glutathione biosynthesis (cystathionine synthase genes, log_2_FC = 3.7). Correlation analysis identified an o*mpR*–s*igV* axis through which *E. faecalis* discriminates mineral particle size, with nanoscale particles eliciting stronger transcriptional responses than their larger counterparts. This stress response additionally upregulated virulence-associated genes and antibiotic resistance genes without direct antimicrobial selection pressure. These findings suggest that non-nutritive minerals shape microbial physiology through physical and surface-chemical cues independent of their nutritional value, highlighting the need for further exploration of the non-nutritional functions of minerals in microbial ecology.

**IMPORTANCE:**

Even in the absence of utilizable nutrients, non-nutritive minerals such as Al_2_O_3_ and SiO_2_ can profoundly influence the transcriptional responses of *Enterococcus faecalis*. Using transcriptomic sequencing, we show that these inert minerals regulate microbial transcription, enhancing iron acquisition, oxidative stress repair, pathogenicity, and antibiotic resistance. Mineral-mediated transcriptional control is driven primarily by physical contact, surface chemistry, and particle size sensing, rather than conventional metabolic interactions. These findings identify inert minerals as signaling molecules that actively modulate microbial transcription, playing a proactive role in microbial evolution and environmental adaptation. This study redefines inert minerals as active carriers of transcriptional regulation, filling a critical gap in geomicrobiology and providing new insights into microbial environmental responses, biogeochemical cycling, and the mechanisms underlying microbial functional evolution and maintenance.

## INTRODUCTION

Minerals govern fundamental aspects of microbial life. They supply essential metal cofactors (e.g., iron, manganese, and molybdenum) for enzymatic reactions and redox processes (1, 2). Iron–sulfur cluster-containing enzymes are critical for glycolysis and oxidative phosphorylation; manganese participates in oxidative metabolism; and molybdenum catalyzes nitrogen fixation (3–5). Minerals also serve as electron donors or acceptors in respiration, exemplified by bacteria that reduce ferric iron or convert sulfate to hydrogen sulfide (6). Conversely, they can impose toxicity. Tungsten, for instance, suppresses biomass accumulation by inhibiting anabolic processes, while lead and lanthanum inhibit the citric acid cycle (7, 8).

To acquire essential nutrients or energy, microbes have evolved sophisticated sensing and response mechanisms tailored to specific minerals. Chemotaxis enables directed movement toward or away from mineral gradients (9, 10), while two-component systems coordinate adaptive responses to metal availability through sensor kinases and response regulators (11, 12). Alternative σ factors direct global transcriptional reprogramming under specific conditions (13, 14). When facing nutrient limitation, bacteria deploy specialized acquisition strategies such as siderophore secretion for iron sequestration (15) or surface modification to enhance mineral solubilization (16). Together, these mechanisms enable microorganisms to exploit diverse mineral resources across environments, contributing to the ecological success and evolutionary diversification of the microbial world.

However, this depth of understanding does not extend to non-nutritive minerals, which provide neither nutrients nor redox potential. Conventionally regarded as biochemically inert, these minerals have been studied primarily for their passive physical roles, such as facilitating biofilm formation and microbial attachment, altering local hydrodynamics to impact motility, offering microscale refuges against desiccation and radiation (17–20), and sequestering organic carbon in soils to create zones of nutrient limitation (21). Since sensing mechanisms for nutritive minerals are fundamentally coupled to their supply signals, non-nutritive minerals, which lack any such input, would not be expected to engage dedicated regulatory machinery. Yet, physical contact with mineral particles in the absence of nutrient exchange or chemical toxicity has been documented to trigger changes in stress response gene expression and metabolic activity (22, 23), indicating microorganisms may possess mineral-sensing mechanisms operating independently of nutritional homeostasis. Our previous work has also shown that minerals actively reshape community structure in nutrient-rich media, driving functional differentiation of the rare biosphere and upregulating genes associated with antibiotic resistance and organic matter degradation (24, 25). This mismatch between expectation and observation raises key questions about whether microorganisms sense non-nutritive minerals as environmental signals, discriminate among minerals by physical properties, and gain adaptive advantages from such responses in mineral-rich environments.

To address these questions, we investigated the transcriptional responses of *Enterococcus faecalis* to non-nutritive mineral exposure. We selected this facultative anaerobic gram-positive bacterium for its environmental ubiquity and robustness. Its compact genome, well-characterized regulatory networks, and extensive use of two-component systems and stress-responsive σ factors make it particularly suited for interrogating transcriptional responses to environmental changes (26–28). Meanwhile, *E. faecalis* is not only a prominent opportunistic pathogen but also a ubiquitous environmental bacterium widely distributed across soils, sediments, natural waters, vegetation, and animal-associated habitats (29, 30). Its remarkable tolerance to diverse environmental stressors facilitates its long-term persistence and colonization across a broad range of ecological niches, making it an ecologically relevant model for studying microbial environmental adaptation. Transcriptomic analysis provides a direct approach to capturing the immediate regulatory responses of *E. faecalis* to mineral exposure by quantifying expressed functional genes. Compared with genome sequencing, which reflects the microorganism’s genetic potential, transcriptomics reveals the active molecular processes underlying microbial adaptation to environmental stimuli (31, 32). We exposed the microorganism to aluminum oxide (Al_2_O_3_) and silicon dioxide (SiO_2_)—minerals chosen for their environmental abundance, chemical inertness under physiological conditions, and lack of bioavailable nutrients (33)—at defined particle sizes. We hypothesized that (i) these minerals act as environmental signals that modulate microbial growth and gene expression despite lacking metabolic utility, and (ii) cellular responses are tuned to mineral surface properties and particle size. By defining the transcriptomic signature of these interactions, we provide evidence that non-nutritive minerals function as informational components of the microbial environment, revealing how microorganisms integrate abiotic physical cues into their regulatory networks.

## RESULTS

### Mineral adhesion and differential growth effects

**Fig 1:**
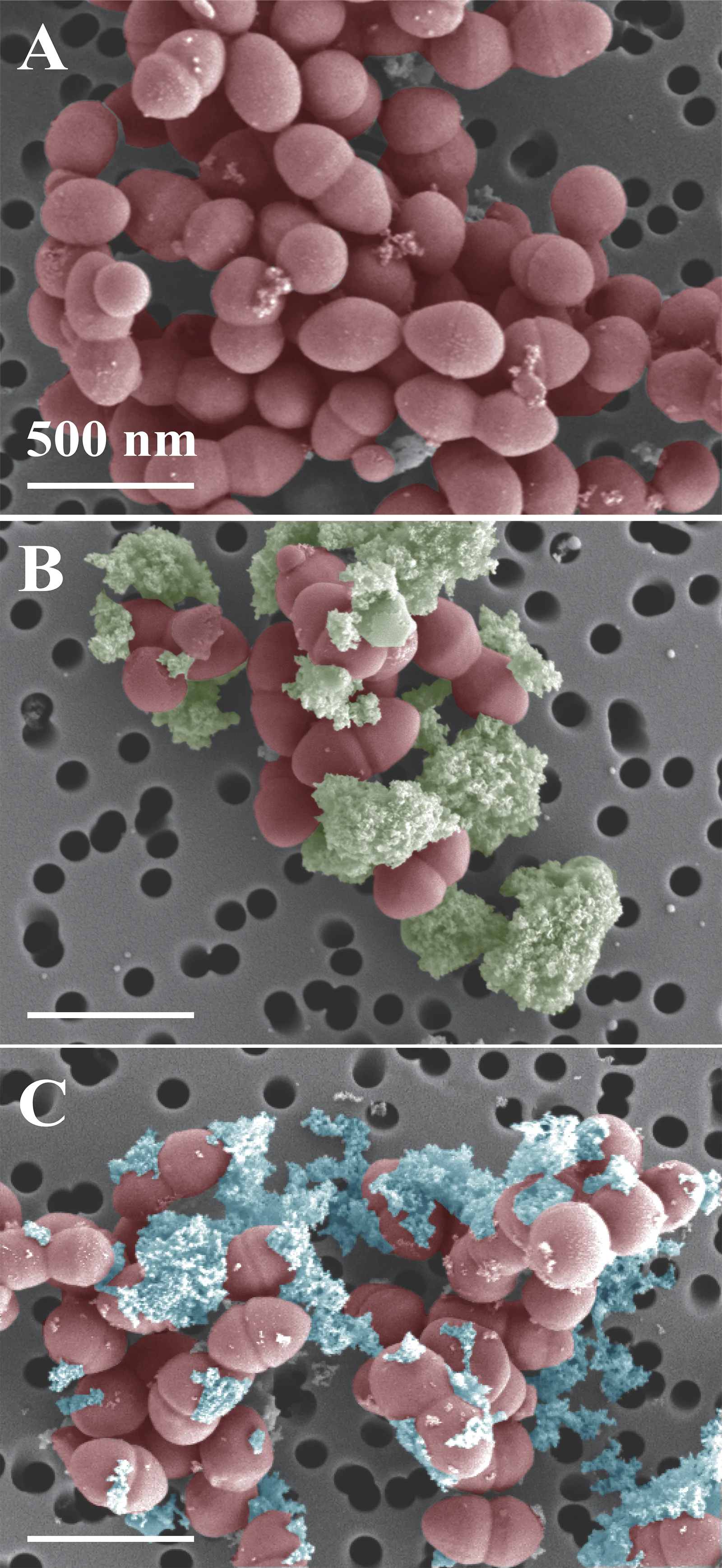
SEM microphotographs showing *E. faecalis* cells (**A**) collected from pristine LB medium and treated with (**B**) 50 nm Al_2_O_3_ and (**C**) 12 nm SiO_2_ particles. Notice the stability of cell morphology throughout the experiments and the formation of cell–mineral complexes under treatment. To facilitate visual identification, false color labeling was applied to cells and mineral particles, with *E. faecalis* rendered in red, Al_2_O_3_ in green, and SiO_2_ in blue.

Electron microscopy confirmed that both Al_2_O_3_ and SiO_2_ particles adhered to *E. faecalis* cell membranes, forming mineral–microbe complexes (Fig. 1; Fig. S1). Neither mineral treatment caused observable cell lysis or morphological alterations. However, cell viability assays (Fig. S2A) showed that Al_N increased CFU counts by ~24%, whereas Si_N1 decreased them by ~19% relative to CK. Across all particle sizes, Al_2_O_3_ increased growth by an average of ~16%, while SiO_2_ reduced it by ~11%.

### Transcriptomic response to mineral exposure

**Fig 2:**
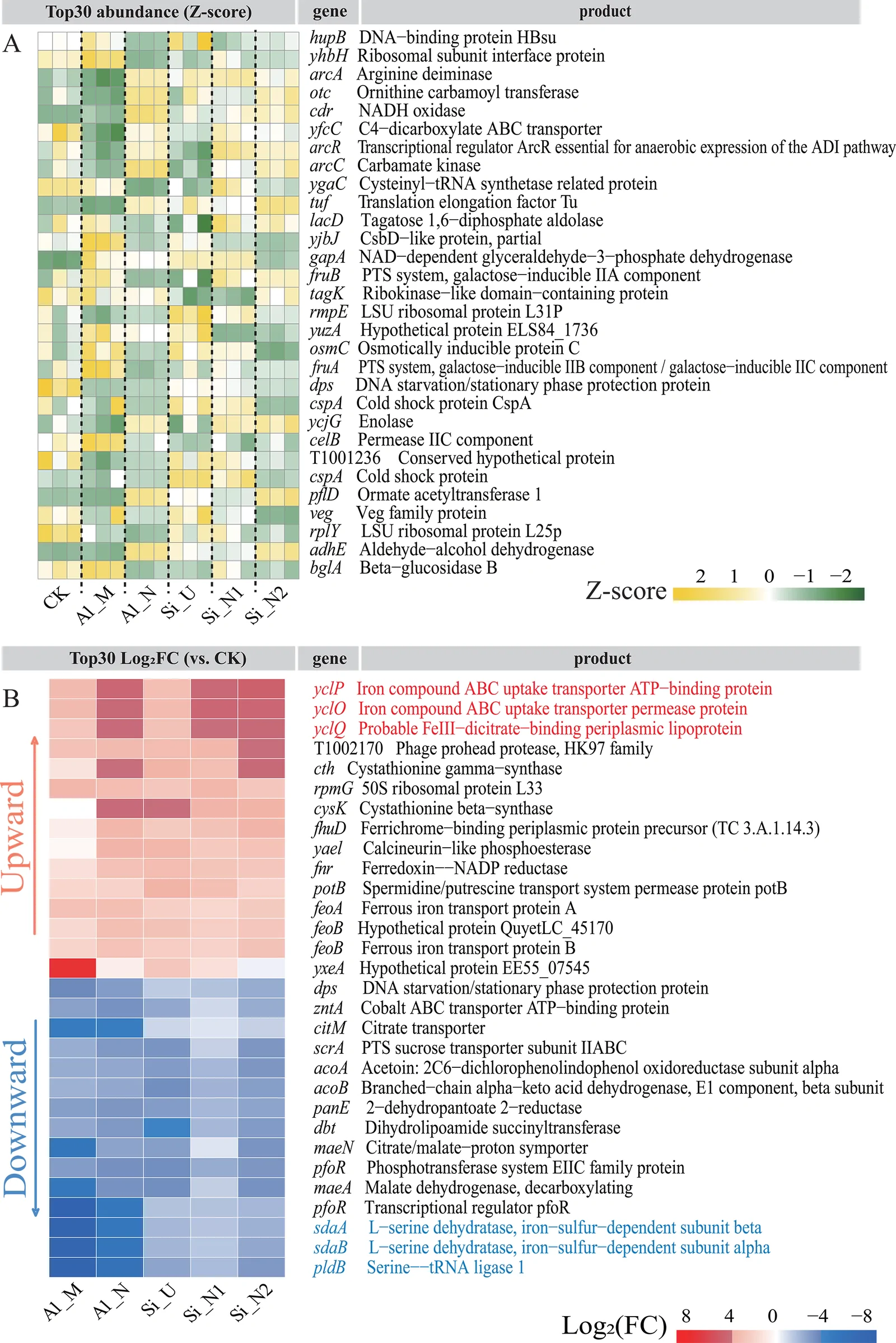
Heatmap showing genes with the highest (**A**) transcriptional abundance and (**B**) fold changes in *E. faecalis* under Al_2_O_3_ and SiO_2_ treatments. Color-coded squares for each treatment in panel A correspond to three replicates. Notice in panel B that iron uptake (text in red) and serine metabolism (text in blue) exhibited the most pronounced response following Al_2_O_3_ and SiO_2_ treatment. Color intensity in panels A and B reflects *Z*-scores and log_2_FC values, respectively.

**Fig 3:**
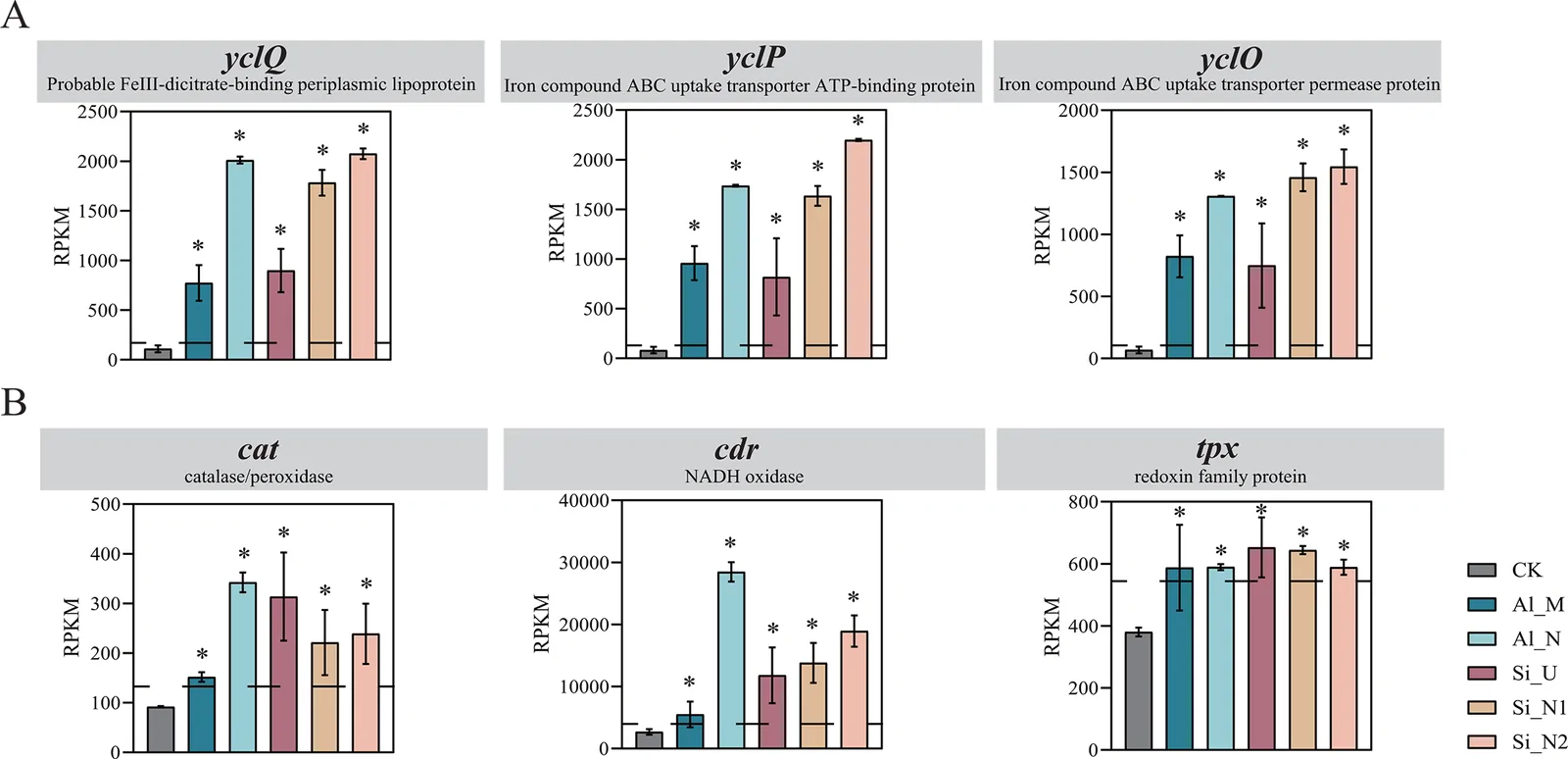
Effects of Al_2_O_3_ and SiO_2_ on gene transcription relative to (**A**) iron uptake and (**B**) oxidative stress reduction. Compared with the CK group, asterisks (*) represent statistical significance (|log_2_FC| > 0.5 and FDR < 0.05), and the dotted line represents a |log_2_FC| = 0.5 cutoff. Data are shown as mean ± standard deviation of three independent biological replicates (*n* = 3).

Transcriptome sequencing revealed widespread transcriptional changes following mineral exposure, with hundreds of differentially expressed genes (DEGs) (Fig. S2B). Kyoto Encyclopedia of Genes and Genomes (KEGG) enrichment analysis identified affected pathways spanning metabolism, environmental signal processing, and genetic information processing, with amino acid, carbohydrate, and lipid metabolism as well as signal transduction prominently represented (Fig. S3). Among the genes with the highest transcriptional abundance (Fig. 2), NADH oxidase showed notable upregulation (log_2_FC = 2.56), while DNA starvation/stationary-phase protection protein (Dps) was strongly repressed (log_2_FC = −2.2). Iron uptake genes (*yclQ*, *yclP*, and *yclO*) exhibited the strongest overall response (log_2_FC from 3.8 to 4.2), with smaller particles eliciting greater induction. Serine metabolism genes (*sdaA* and *sdaB*) showed comparably strong repression, decreasing (log_2_FC = −2.6 and −2.7, respectively). Oxidative stress-related genes were also significantly upregulated: *cat* (log_2_FC = 1.5), *cdr* (log_2_FC = 2.5), and *tpx* (log_2_FC = 0.7) (Fig. 3).

Mineral treatments additionally altered virulence and antibiotic resistance gene expression. Virulence factor analysis (Fig. S7) showed high relative abundance in categories of antiphagocytosis (34%), adherence (21%), immune evasion (12%), and biofilm formation (9%). Relative to CK, mineral exposure upregulated adherence (log_2_FC = 0.7) and invasion (log_2_FC = 0.6) genes but downregulated secretion system genes (log_2_FC = −0.5). Most treatments (except Al_M) also significantly upregulated antibiotic resistance genes (Fig. S8).

### Effects on two-component systems and σ factors

**Fig 4:**
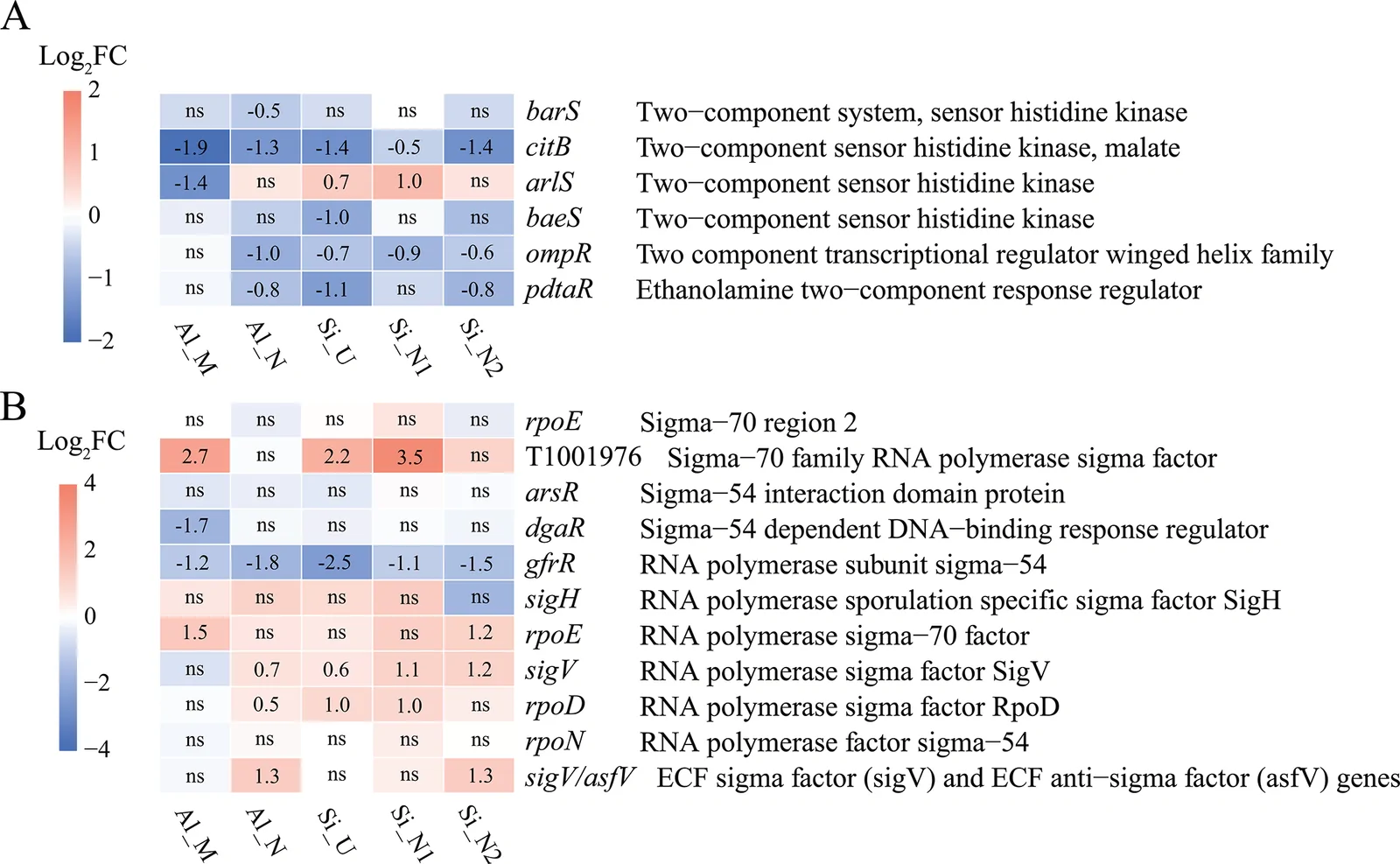
Heatmap showing the effects of Al_2_O_3_ and SiO_2_ on gene transcription in (**A**) two-component systems and (**B**) σ factors. Boxes with numbers show fold changes for genes with significant (|log_2_FC| > 0.5 and FDR < 0.05) differential expression (vs CK); boxes with “ns” denote non-significant differences. Color temperature (warm vs cool) indicates upregulation and downregulation, respectively.

**Fig 5:**
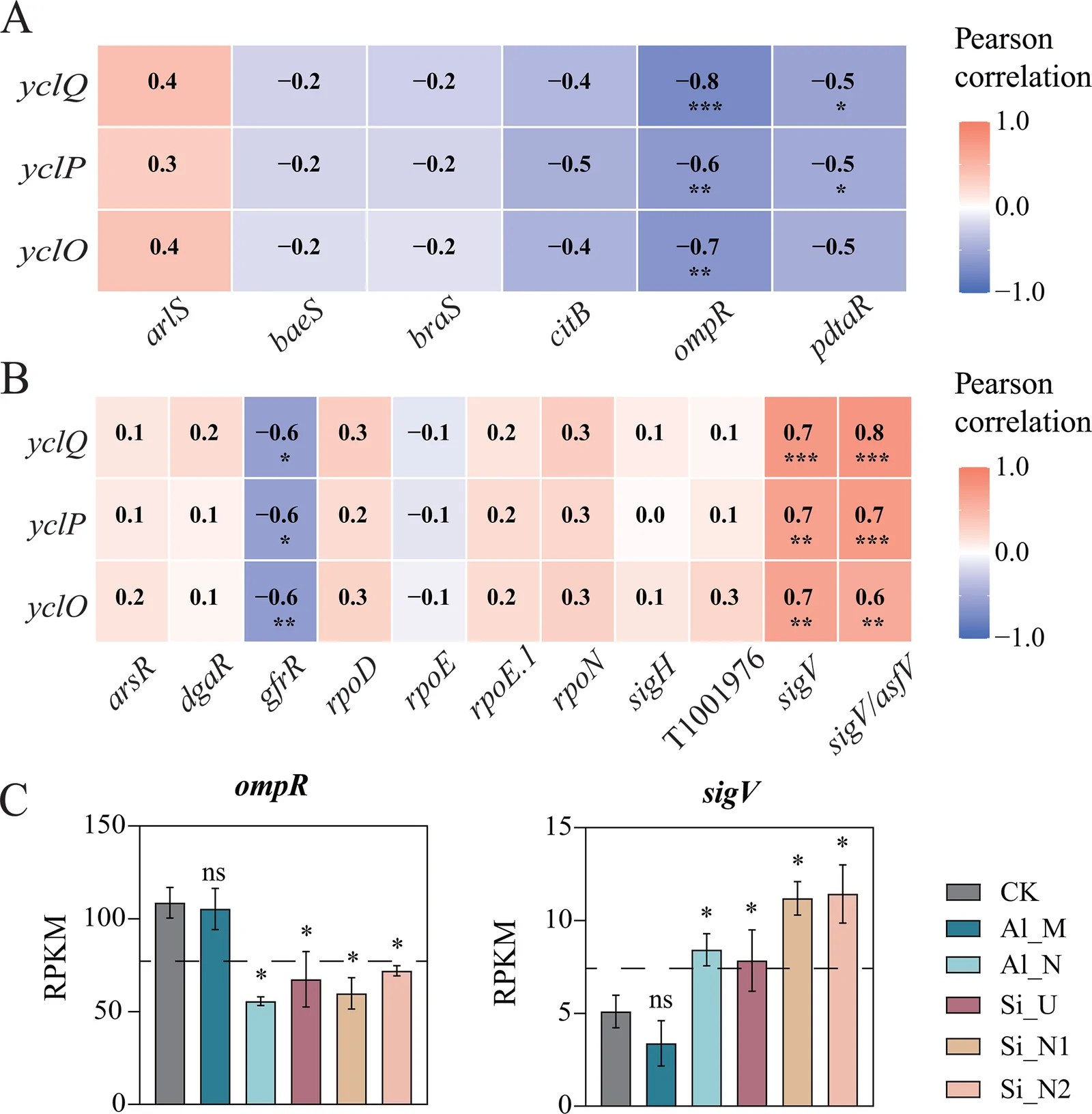
Correlation analysis of (**A**) two-component systems and (**B**) σ factors to identify (**C**) potential regulators of iron uptake genes. In panels A and B, *, **, and *** indicate significance at the 0.05, 0.01, and 0.001 probability levels, respectively. Values in boxes denote correlation coefficients. Compared with the CK group, in panel C, asterisks (*) represent statistical significance (|log_2_FC| > 0.5 and FDR < 0.05); ns indicates non-significance, and the dotted line denotes a |log_2_FC| cutoff of 0.5. Data are shown as mean ± standard deviation of three independent biological replicates (*n* = 3).

Mineral exposure downregulated most TCS genes (Fig. 4A). Specifically, c*itB* decreased significantly (log_2_FC = −1.2), while *pdtaR* and o*mpR* showed modest decreases (log_2_FC = −0.6 for both). In contrast, with the exception of the medium-sized Al_M treatment, mineral addition significantly upregulated the extracytoplasmic function (ECF) σ factor s*igV* (log_2_FC = 0.7) and the housekeeping σ factor r*poD* (log_2_FC = 0.6) while downregulating the regulator *gfrR* (log_2_FC = −1.5) (Fig. 4B). Correlation analysis identified TCS o*mpR* and σ factor s*igV* as key predictors of iron uptake gene expression, with their transcriptional patterns showing strong dependence on mineral particle size (Fig. 5). Additionally, mineral exposure altered quorum sensing gene expression, with coarse-grained minerals increasing transcript abundance by 25% relative to fine-grained minerals (Fig. S4A).

### Response of pyruvate and amino acid metabolism

Mineral treatments increased L-lactate dehydrogenase (*ldh*) gene abundance with a log_2_FC of approximately 2.38 relative to CK (Fig. S5). Furthermore, genes encoding the pyruvate dehydrogenase E1 component (α- and β-subunits) were upregulated, with log_2_FC values of 0.55 and 0.97, respectively, except in Al_M (Fig. S5). Serine dehydratase α- and β-subunits were strongly repressed, with log_2_FC values of −2.74 and −2.56, respectively (Fig. S6). Among iron-dependent enzymes, pyruvate ferredoxin oxidoreductase and ferredoxin (flavodoxin) oxidoreductase exhibited log_2_FC values of −0.45 and 1.07, respectively (Fig.S5). Genes associated with the uptake and biosynthesis of glutamate, cysteine, and glycine (*glnP*, cystathionine β-synthase, glutamate permease, glycine cleavage H-protein, and cystathionine γ-synthase) were upregulated, with log_2_FC values ranging from 1.04 to 4.02 in all treatments except Al_M (Fig. S6).

## DISCUSSION

With hundreds of differentially expressed genes across treatments (Fig. S2), mineral exposure triggered comprehensive physiological remodeling spanning metabolism, signal transduction, and genetic information processing. This pattern is characteristic of general stress responses in enterococci and other Firmicutes (Table S2) (34, 35). These transcriptomic data provide compelling evidence that Al_2_O_3_ and SiO_2_ function not only as passive physical substrates but also as environmental cues capable of inducing widespread transcriptional reprogramming in *E. faecalis*.

### Mineral-induced oxidative stress and the iron paradox

Central to this response is the induction of oxidative stress. The significant upregulation of antioxidant-related genes, including *cat* (log_2_FC = 1.5), *cdr* (log_2_FC = 2.5), and *tpx* (log_2_FC = 0.7) (Fig. 3), indicates a robust cellular response to reactive oxygen species (ROS). This is further supported by the increase in NADH oxidase expression (log_2_FC = 2.6), which catalyzes the reduction of O_2_ to H_2_O or H_2_O_2_ using NADH as an electron donor and plays a dual role in oxidative stress management and NAD^+^ regeneration (36). Reactive oxygen generation (including H_2_O_2_, O_2_^−^, and OH radical-like species) at mineral surfaces is a general phenomenon observed for oxides, silicates, and sulfides (37, 38). For Al_2_O_3_ and SiO_2_, it was specifically shown that the presence of hydroxyl radicals, superoxide, and hydrogen peroxide occurs through surface defect sites and mechanoradical formation (39, 40). The adherence of mineral particles to cell membranes, as confirmed by our electron microscopy data (Fig. 1), places these ROS-generating surfaces in direct proximity to cellular components, maximizing oxidative damage potential.

It is noteworthy that despite this extensive internal transcriptional reprogramming and the proximity of ROS-generating mineral surfaces, electron microscopy revealed no significant alterations in cell morphology (Fig. 1). This decoupling of internal physiological state and external structure indicates that the ROS generated by minerals act as sublethal stressors rather than acute toxins. The robust upregulation of cell envelope stress responses (mediated by s*igV*) and antioxidant systems appears sufficient to preserve physical integrity, albeit likely at a metabolic cost.

This oxidative stress presents a striking physiological paradox when viewed alongside the iron metabolism results. We refer to this as a “paradox” because bacteria typically repress iron uptake during oxidative stress to prevent the Fenton reaction (Fe^2+^ + H_2_O_2_ → Fe^3+^ + •OH + OH^−^), which would exacerbate ROS damage. In addition, LB medium is a nutrient-rich condition that is fully sufficient to meet the iron requirements of *Enterococcus faecalis*. However, *E. faecalis* upregulated (log_2_FC range from 3.8 to 4.2) iron uptake genes (*yclQ*, *yclP*, and *yclO*) while simultaneously upregulating ROS defenses (Fig. 3). Although the pleiotropic effects of s*igV* cannot be ruled out, this drastic upregulation more than likely points to a “starvation-in-the-midst-of-plenty” scenario driven by two complementary mechanisms.

1. Electrostatic adsorption: the point-of-zero-charge pH difference of Al_2_O_3_ (~8) and SiO_2_ (~4) leads to differential interfacial reactions at culture pH, either facilitating or hindering bacterial growth. Negatively charged SiO_2_ may adsorb cationic nutrients such as iron and other metal cofactors, effectively creating localized zones of nutrient depletion (41, 42) by sequestering them from microbial uptake (43). In addition, electrostatic repulsion between the SiO_2_ particles and the bacterial cell surface may further impede growth by inhibiting biofilm formation. Positively charged Al_2_O_3_ surfaces, by contrast, are not subject to either of these effects and may instead promote cell attachment and nutrient availability. The contrasting effects of growth promotion by Al_2_O_3_ against inhibition by SiO_2_, particularly for nanoparticles (24% increase vs ~19% decline), may partly reflect these differences.
2. Steric hindrance: the formation of mineral–microbe complexes (Fig. 1) likely compromises the function of membrane transport proteins and the ensuing transmembrane nutrient uptake. The stronger iron-starvation response elicited by smaller particles supports this hypothesis as particles with greater specific surface area can more tightly encapsulate the bacterial cell, creating a more severe physical barrier to nutrient diffusion and forcing the cell to hyper-activate high-affinity transport systems (44).

The decrease in *dps* expression (log_2_FC = −2.2) further reinforces this physiological paradox, effectively linking the oxidative stress and iron limitation responses. Dps typically sequesters intracellular iron to prevent Fenton chemistry and protect DNA from oxidative damage (45). Its downregulation suggests a regulatory trade-off where the cell prioritizes free iron availability for critical metalloenzymes over DNA protection. However, this creates a precarious feedback loop: increased intracellular free iron (via uptake upregulation and *dps* repression) combined with mineral-generated ROS heightens the risk of Fenton-mediated damage. This necessitates the simultaneous, robust upregulation of catalase and peroxidase (Fig. 3) as a compensatory mechanism to scavenge ROS before they can react with the bioavailable iron pool.

A potential concern is whether the observed transcriptional responses in Al_2_O_3_ treatments reflect aluminum toxicity rather than cell–mineral interactions. We consider this unlikely for two reasons. First, Al_2_O_3_ dissolution at circum-neutral pH is thermodynamically constrained by gibbsite solubility (Ksp ≈ 10^−33.5^), which limits free Al^3+^ activity to approximately 10^−11^ M at pH 6.5. This concentration is roughly eight orders of magnitude below the minimum inhibitory concentration of ~1 mM Al^3+^ reported for bacterial growth inhibition (46, 47). Furthermore, at pH 6.5, dissolved aluminum exists predominantly as hydroxyl–Al complexes [Al(OH)^2+^ and Al(OH)_2_^+^] rather than the free Al^3+^ ion responsible for biological toxicity, further reducing effective bioavailability (48). Second, if aluminum toxicity were a contributing factor, one would expect Al_2_O_3_-treated cells to exhibit impaired growth relative to the mineral-free control. Instead, Al_2_O_3_ treatments consistently promoted growth, with Al_N increasing CFU counts by 23%. This growth-promoting effect is more consistent with a surface charge-mediated nutrient availability mechanism described above than with ionic toxicity. These lines of evidence collectively argue against aluminum toxicity as a driver of the transcriptional responses observed in Al_2_O_3_ treatments.

### Transcriptional integration of particle size via TCS, σ factors, and quorum sensing

The downregulation of TCS genes c*itB* (log_2_FC = −1.2), p*dta*R (log_2_FC = −0.6), and o*mpR* (log_2_FC = −0.6) (Fig. 4A) suggests a “dampening” of canonical sensing, representing a threshold recalibration to prevent overstimulation under mineral stress (49, 50). Conversely, the upregulation of σ factors s*igV* (log_2_FC = 0.7) and r*poD* (log_2_FC = 0.6) (Fig. 4B) indicates targeted activation of cell envelope stress responses and simultaneous maintenance of growth-oriented transcription, analogous to a “growth-defense balance” strategy (51). The significant change in s*igV* (associated with cell envelope stress in *E. faecalis*) suggests sensing of subtle membrane perturbations prior to visible morphological damage.

The millimeter-sized Al_2_O_3_ treatment frequently deviated from these trends, showing minimal activation of s*igV* or r*poD*. This can be attributed to physical scale and contact probability. Unlike nanoparticles that possess immense specific surface area and can form intimate “encapsulating” complexes with cells, millimeter-sized particles present a minimal surface interface relative to their mass and rapidly settle out of suspension. Consequently, the effective “dose” of mineral surface stress experienced by the planktonic bacterial population is negligible. This lack of sustained physical contact explains why Al_M failed to trigger the threshold-dependent stress responses (such as s*igV* upregulation or ARG induction) that were prominently activated by the highly reactive, high-surface-area nanometer and micrometer particles. Crucially, the millimeter-scale Al_2_O_3_ treatment (Al_M) serves as a valuable internal control, enabling us to differentiate true surface-mediated interactions from the non-specific, generic effects induced by the mere introduction of solid material. The paucity of significant DEGs in the Al_M group relative to CK demonstrates that the observed microbial responses were not a generalized artifact of particle addition. Instead, this internal validation strongly reinforces our interpretation that the transcriptomic reconfigurations are specifically dependent on sustained, active cell–surface contacts, which are themselves tightly governed by particle size.

Notably, correlation analysis (Fig. 5) identified TCS o*mpR* and σ factor s*igV* as key predictors of iron uptake gene transcription, with expression patterns dependent on particle size. Small particles repressed o*mpR* but strongly induced s*igV*. This indicates that *E. faecalis* integrates the physical stress of “encapsulation” by fine particles into a specific transcriptional program. While the interaction between bacterial cells and mineral particles is fundamentally physicochemical, the differential activation of the *ompR–sigV* axis suggests that these regulators may participate in the cellular response to different degrees of mineral surface contact. These associations allow us to propose a tentative mechanistic hypothesis regarding how mineral particle size might influence cell-envelope-associated signaling. When bacteria colonize mineral surfaces, the localized contact creates size-dependent physical constraints, such as mechanical shear, local membrane deformation, or altered surface-charge topography. Because the *ompR* system is traditionally sensitive to osmolarity and envelope stress, and the ECF σ factor *sigV* is primarily dedicated to monitoring cell envelope integrity (52, 53), we hypothesize that different particle sizes exert distinct degrees of physical perturbation on the cell envelope. This microenvironmental friction may trigger a coordinated adaptive program, wherein the cell reconfigures its envelope homeostasis (*sigV*) while simultaneously rewiring its downstream metabolic networks (*ompR*) to adapt to the physical confinement. However, this hypothesis is based on correlative transcriptomic evidence and should be validated through targeted genetic and biochemical studies.

Beyond their direct transcriptional effects, minerals also disrupt cell-to-cell communication by adsorbing autoinducer molecules. The alteration of quorum sensing genes, particularly the 25% increase in expression under coarse-grained mineral treatment relative to fine-grained minerals (Fig. S4A), likely reflects surface area-dependent adsorption. Fine-grained minerals, with their vastly higher specific surface area, may adsorb and sequester autoinducer signaling molecules more effectively than coarse minerals (54), thereby dampening the population density signal and suppressing quorum sensing regulons.

### Metabolic reprogramming toward redox balance and antioxidant biosynthesis

To survive mineral-induced oxidative stress and iron perturbation, *E. faecalis* orchestrates a coordinated metabolic shift from biomass accumulation to defense, simultaneously rerouting pyruvate metabolism to maintain redox balance and channeling serine flux toward cysteine and glutathione biosynthesis.

#### Redox balance and energy maintenance

The massive upregulation of NADH oxidase (log_2_FC = 2.6) functions as the physiological linchpin connecting oxidative stress defense to metabolic reprogramming (55, 56). While this enzyme serves a primary protective role by reducing intracellular oxygen (detoxifying ROS), this activity imposes a severe drain on the NADH pool. Consequently, the increase in *ldh* gene abundance (log_2_FC = 2.4) represents a critical compensatory adaptation (Fig. S5F). By favoring the reverse reaction (lactate oxidation to pyruvate) within the reversible pyruvate–lactate conversion, LDH replenishes the NADH pool, thereby sustaining microbial survival and counterbalancing the NADH-depleting pull of NADH oxidase (57). This elegant coupling ensures that the cell can maintain high-throughput ROS detoxification without stalling the energy-generating pathways essential for repair. Furthermore, the increased pyruvate availability, together with elevated pyruvate dehydrogenase expression (log_2_FC range from 0.6 to 1.0, except Al_M), promotes pyruvate entry into the TCA cycle, supplying energy and biosynthetic precursors for oxidative damage repair. It should be noted that the inferred directionality of *ldh*-associated metabolic flux is based on transcriptional evidence and thermodynamic reasoning rather than direct metabolic flux measurements. Therefore, the observed changes in *ldh* expression should be interpreted as transcriptional changes consistent with potential pyruvate–lactate flux redirection, rather than as evidence of demonstrated metabolic rerouting. Future investigations employing stable isotope tracing or quantitative metabolomics will be crucial to confirm whether these transcriptional responses translate into concrete metabolic flux reconfigurations.

#### Biosynthetic rerouting for antioxidants

The noticeable repression of serine dehydratase (log_2_FC = −2.7) represents a strategic metabolic shift in which serine flux is being redirected from energy catabolism toward cysteine biosynthesis. This is evidenced by the massive upregulation of cystathionine β-synthase and γ-synthase (log_2_FC = 3.7). Cysteine is the rate-limiting precursor for low-molecular-weight thiols (such as glutathione, critical for ROS detoxification [58]) and an essential building block for iron–sulfur clusters needed for repairing oxidative damage (59). The simultaneous upregulation of glutamate and glycine metabolism genes (*glnP*, log_2_FC = 1.6; glutamate permease, log_2_FC = 1.0; and glycine cleavage H-protein, log_2_FC = 1.2) (Fig. S6) reflects both enhanced extracellular uptake and intracellular biosynthesis of all three glutathione precursors (glutamate, cysteine, and glycine). Together, the bacterium effectively reengineers its nitrogen flux to synthesize the antioxidant “ammunition” needed to combat mineral-induced ROS.

#### Iron conservation

The differential response of iron-dependent enzymes (Fig. S5), i.e., the switch from iron-dependent pyruvate ferredoxin oxidoreductase (log_2_FC = −0.5) to iron-independent flavodoxin oxidoreductase (log_2_FC = 1.1) further illustrates cellular adaptation to altered iron availability. This enzyme substitution is consistent with a canonical iron-sparing response (60), a classic strategy across diverse organisms, providing further evidence for the iron-perturbing effects of mineral exposure.

### Unintended consequences of enhanced virulence and antibiotic resistance

The presence of mineral stress appears to activate pathways associated with “cross-protection,” whereby generalized stress responses inadvertently prime the bacterium for enhanced pathogenicity. Among the identified virulence factors, those related to host-defense avoidance were most abundantly represented, including antiphagocytosis (34%) and immune evasion (12%), followed by adherence (21%) and biofilm formation (9%) (Fig. S7). The concurrent upregulation of adherence genes (log_2_FC = 0.7) and downregulation of certain secretion components suggest a remodeling of the cell surface toward an adhesion-competent state. Rather than a specific host-mimicry signal, the mineral interface likely triggers a generalized envelope stress response that incidentally overlaps with adhesion programs, a response potentially mediated by s*igV*, which has been implicated in both cell envelope stress and surface-associated functions in *E. faecalis* (26, 61). The representation of biofilm formation genes among identified virulence factors (9%) may further reflect the natural progression from mineral surface adhesion to structured community development, as mineral particles provide the solid interface and spatial heterogeneity conducive to biofilm establishment. It should be noted that this overlap is inferred from transcriptomic patterns rather than functional adhesion or invasion assays, and whether mineral-induced gene expression translates to enhanced host-cell binding capacity remains to be tested directly.

Furthermore, our data (Fig. S8) suggest that mineral-induced oxidative stress may promote antibiotic resistance through upregulation of ARGs and activation of efflux systems. Mineral exposure likely acts as a sublethal stressor to activate these responses, potentially rendering the bacteria less susceptible to subsequent antimicrobial therapies. In this context, mineral presence may contribute to conditions favoring the persistence and clinical recalcitrance of these opportunistic pathogens.

### Geomicrobiological implications

The ability of *E. faecalis* to discriminate both mineral chemistry and particle dimension, independent of nutritional or energetic value, challenges the conventional view of non-nutritive minerals as passive substrates and suggests that their physical and surface-chemical properties constitute environmental information that bacteria actively integrate into regulatory decisions. This capacity carries two broad evolutionary implications. First, physical mineral sensing may represent a regulatory primitive predating chemical nutrient sensing. In the mineral-dominated environments of early Earth, abiotic physical contact may have been among the earliest selective pressures shaping the architecture of two-component systems and σ factor networks, positioning mineral–microbe signaling at the evolutionary origin of environmental sensing itself. Second, the concurrent induction of ARGs, virulence programs, and broad transcriptional reprogramming under sublethal mineral stress has direct implications for stress-driven evolvability, representing the emerging principle that environmental stressors accelerate evolutionary diversification through phenotypic plasticity, error-prone repair, and horizontal gene transfer competence. Mineral-rich environments, ubiquitous across natural and anthropogenic settings alike, may therefore function as unrecognized evolutionary hotspots where resistance and pathogenicity traits are inadvertently but repeatedly selected, with significant consequences for the long-term trajectory of opportunistic pathogen evolution.

### Conclusions

Exposure to non-nutritive Al_2_O_3_ and SiO_2_ induced extensive transcriptional reprogramming in *E. faecalis*, demonstrating that chemically inert minerals function as environmental signals independent of nutritional or energetic input. Mineral addition triggered simultaneous upregulation of iron acquisition genes and oxidative stress defenses, representing a transcriptional signature suggestive of an “iron paradox.” This response may be driven by mineral-induced nutrient sequestration and steric hindrance of membrane transporters rather than ionic toxicity, as dissolved Al^3+^ at experimental pH falls orders of magnitude below bacterial toxicity thresholds. In response, the bacterium shifted metabolism from biomass accumulation to defense, rerouting serine flux toward cysteine and glutathione biosynthesis while sustaining redox balance via enhanced *ldh* and pyruvate dehydrogenase activity.

Particle size was integrated as a discrete regulatory input through an o*mpR*–s*igV* regulatory axis, translating fine particle encapsulation into a targeted envelope stress response and modulating quorum sensing through surface area-dependent autoinducer sequestration. These adaptations collectively produced a cross-protection effect, inadvertently priming the bacterium for pathogenicity through upregulation of adherence, biofilm formation, and antibiotic resistance genes, suggesting that mineral-rich environments may function as unrecognized evolutionary hotspots where resistance and virulence traits are repeatedly selected under sublethal physical stress.

These findings advance geomicrobiology by revealing that bacteria actively integrate physical and surface-chemical properties of minerals as environmental information into their regulatory decisions. Physical mineral sensing may represent a regulatory primitive predating chemical nutrient sensing. Future research should employ functional genetic approaches, ROS quantification, metabolic flux analysis, and long-term evolutionary experiments to determine whether these transcriptional changes translate to stable adaptive outcomes and whether physical mineral sensing is broadly conserved across the microbial world.

## MATERIALS AND METHODS

### Experimental materials

Aluminum oxide (Al_2_O_3_) and silicon dioxide (SiO_2_) were purchased from Sigma-Aldrich Corporation (St. Louis, MO, USA). Al_2_O_3_ was obtained in two sizes: 3 mm (Al_M) and 50 nm (Al_N), while SiO_2_ was obtained in three sizes: 44 µm (Si_U), 12 nm (Si_N1), and 200 nm (Si_N2). Morphologically, Al_M and Si_N2 are spherical, with the remaining materials are irregular. Detailed product specifications and catalog numbers are provided in Table S1.

In this study, *Enterococcus faecalis* strain HHT-1, isolated from agricultural field soil, was used as the experimental strain. Its genome assembly is available in the NCBI GenBank database under accession number GCA_044706835.1. All *E. faecalis* cultures were grown in LB medium. The LB medium used in this experiment contained the following ingredients: 10 g tryptone, 5 g yeast extract, 10 g NaCl, and 1 L distilled water. The pH of the LB medium was adjusted to 6.5. Since the nutrient composition of LB medium can vary, depending on the manufacturer of tryptone and yeast extract, we selected the classic OXOID brand to ensure that the medium used in this study is consistent with that commonly used in most laboratories.

### Mineral–microbe co-culture experiment

*E. faecalis* was subcultured in LB liquid medium and inoculated at 1% (vol/vol) during the logarithmic growth phase into 250 mL conical flasks containing 50 mL LB medium. For each mineral treatment, 0.1 g of Al_M, Al_N, Si_U, Si_N1, or Si_N2 was added to separate flasks. A mineral-free control (CK) was included. All treatments were performed in triplicate and incubated at 30°C with shaking at 150 rpm. Mineral concentrations ranging from 0.5 to 50 g/L have been widely utilized across various experimental systems (62, 63). To minimize the potential confounding nutritional effects (e.g., nutrient release or excessive adsorption) that often occur at higher concentrations, a relatively low mineral concentration (2 g/L) was used throughout this study. Samples were collected during the logarithmic growth phase for viable cell counting, scanning electron microscopy (SEM), and transcriptomic analysis.

### Cell viability and morphological analysis

Viable cell counts were determined by serial dilution plating during the logarithmic growth phase. Briefly, 0.1 mL of bacterial suspension from each treatment was diluted to 10^−5^ with sterile water. A 0.05 mL aliquot of the diluted suspension was spread onto solid LB medium and incubated at 30°C for 24 h. Colonies were enumerated, and viable counts were calculated as follows: CFU per unit volume = (*C*/*V*) × *M*, where *C* is the average colony count, *V* is the volume plated, and *M* is the dilution factor.

Cell morphology was examined by SEM (Zeiss Sigma 500). Samples were fixed in 2.5% (vol/vol) glutaraldehyde in phosphate-buffered saline, dehydrated through a graded ethanol series, dried, and sputter-coated with platinum prior to imaging. For visual distinction, false colors were assigned in Adobe Photoshop (version 2020), with *E. faecalis* in red, Al_2_O_3_ in green, and SiO_2_ in blue.

### RNA extraction and transcriptomic analysis

Total RNA was extracted using the TRIzol Reagent kit (Invitrogen) according to the manufacturer’s protocol. RNA concentration and purity were assessed with a NanoDrop 2000 Spectrophotometer (Thermo Fisher Scientific). Genomic DNA was removed by DNase treatment, and ribosomal RNA was depleted using the Ribo-Zero rRNA depletion kit. RNA integrity and quantity were further evaluated using TBS380 PicoGreen (Invitrogen) and an Agilent 2100 Bioanalyzer (Agilent Technologies, USA). Qualified mRNA was fragmented and used for cDNA library construction. Paired-end sequencing was performed on an Illumina NovaSeq 6000 platform by Biozeron Biotechnology (Shanghai, China).

Raw reads were quality-filtered using Trimmomatic to remove adapter sequences and low-quality bases. Residual ribosomal RNA reads were identified and removed by alignment against the Rfam database using SortMeRNA. Q20 values for all samples exceeded 99%, confirming high sequencing accuracy.

For functional annotation, the unigene sequences were aligned against public databases to assign functional information. Specifically, sequence alignment against the Non-Redundant Protein Database and the Virulence Factors of Pathogenic Bacteria Database was performed using Diamond (version 0.9.22.123) in BLASTP mode, with an *E*-value threshold of 1e^−5^ to ensure the reliability of the annotations (64, 65). KEGG annotation was performed using KofamScan (version 1.2.0) with default parameters, and the Comprehensive Antibiotic Resistance Database was annotated using BLASTP (version 2.0.0+) (66, 67). As a subset of sequences could not be assigned to any annotated gene, we accordingly adopted a provisional naming system (e.g., T1001236, T1002170, and T1001976) and provided functional annotations for these transcripts. The expression levels of non-redundant genes were quantified using reads per kilobase per million mapped reads. DEGs were identified using the edgeR package in R (version 4.3.3). Specifically, the read counts for each gene were obtained using Rockhopper. Differential expression analysis for each gene was then performed using a negative binomial statistical model for hypothesis testing, yielding *P* values for pairwise gene comparisons. Subsequently, the Benjamini–Hochberg procedure was applied for multiple testing correction, and the false discovery rate (FDR) was used to identify statistically significant comparisons between genes, with screening criteria set at |log_2_(FC)| > 0.5 and FDR < 0.05. Given that nutrient-poor minerals were expected to induce relatively subtle transcriptional responses rather than large-scale transcriptional reprogramming, differential expression was defined using a threshold of |log₂FC| > 0.5 and FDR < 0.05. This threshold was selected to improve the detection of biologically meaningful but moderate transcriptional changes while maintaining rigorous statistical control through false discovery rate correction (68–70).

### Statistical analysis

The experiment consisted of six treatment groups, each with three biological replicates (*n* = 3). The treatment effect on the viability of *E. faecalis* during the logarithmic growth phase was assessed using the Kruskal–Wallis test followed by Dunn’s pairwise comparisons with Bonferroni adjustment in SPSS (version 24.0.0). Statistical significance was set at adjusted *P* < 0.05. Pearson correlation analysis was performed using the R (version 4.3.3) packages Hmisc, dplyr, and tidyr for data cleaning, calculation, and statistical testing. The figures in this study were drawn and arranged using GraphPad Prism 8, the ggplot2 package in R (version 4.3.3), and Adobe Illustrator (version 2023).

## Data Availability

The raw sequencing data generated in this study have been deposited in the China National Genomics Data Center under accession number CRA041632.

## SUPPLEMENTAL MATERIAL

Supplemental material aem.00954-26-s0001.pdf Tables S1 and S2; Fig. S1 to S8.

ASM does not own the copyrights to Supplemental Material that may be linked to, or accessed through, an article. The authors have granted ASM a non-exclusive, world-wide license to publish the Supplemental Material files. Please contact the corresponding author directly for reuse.
