# Supplementary material for: The iron paradox and particle size sensing: transcriptomic response of Enterococcus faecalis to non-nutritive minerals
Source: Appl Environ Microbiol. 2026 Aug 19;92(9):e00954-26. doi: 10.1128/aem.00954-26 (PMC13599488; doi:10.1128/aem.00954-26)
Supplement: Supplementary file 1 [file aem.00954-26-s0001.pdf]

The Iron Paradox and Particle Size Sensing: Transcriptomic Response of *Enterococcus faecalis* to Non-Nutritive Minerals

Luhan Li, Jianchao Zhang*, Sumin Qu, Zhiying Zhou, Beibei Wang, Haitao Wu, Zilin Guo, Lin Shi, Bohao Yin, Xiangyu Zhu, Yuebo Wang, H. Henry Teng*

School of Earth System Science, Institute of Surface-Earth System Science, Tianjin University, Tianjin 300072, China

***Corresponding Authors:**

H. Henry Teng* Email: hteng@gwu.edu

Jianchao Zhang* Email: jianchaozhang@tju.edu.cn

Table. S1. Particle Size & Product Number

Mineral	treatment	Size	Shape	Product number	Purity
Al_2O_3	Al_M	3 mm	pellets	414069	
	Al_N	50 nm	nanopowder	544833	
SiO_2	Si_U	44 µm	powder	342890	99.5%
	Si_N1	12 nm	nanopowder	718483	99.8%
	Si_N2	200 nm	mesoporous nanoparticles	748161	

Table. S2 Key Metabolic and Signaling Changes Induced by Minerals

Pathway/Category	Key Genes Affected	Log$_2$FC value (vs. CK)	Implications
Iron Uptake	*yclQ*, *yclP*, *yclO*	3.8 to 4.2	Enhanced iron acquisition, potential overload leading to oxidative stress
Oxidative Stress	*cat*, *cdr*, *tpx*	0.7 to 2.5	ROS defense activation, linked to metal ion catalysis
Serine Metabolism	*sdaA*, *sdaB*	-2.6 to -2.7	Resource shift from amino acids to energy production
Lactate metabolism	*ldh*	2.4	NADH and NAD+ supply
Pyruvate Metabolism	*acoA*, *acoB*	0.5 to 1.0	Accelerated TCA cycle entry for NADH/GSH supply
Amino Acid Metabolism (Glu, Cys, Gly)	*glnH*, *cysK*, etc.	1.0 to 4.0	Increased biosynthesis/uptake for antioxidant precursors
Virulence Factors	Adherence, Immune evasion genes	0.1 to 0.7	Potential increase in pathogenicity, membrane-related
Antibiotic Resistance	Various (e.g., efflux pumps)	Upregulated in most treatments	Enhanced resistance, environmental adaptability
Two-Component Systems	*citB*, *pdtaR*, *ompR*	-0.6 to -1.2	Altered signal perception, particle size sensitivity
Sigma Factors	*sigV*, *rpoD*	0.6 to 0.7	Stress-specific transcriptional regulation

Fig. S1

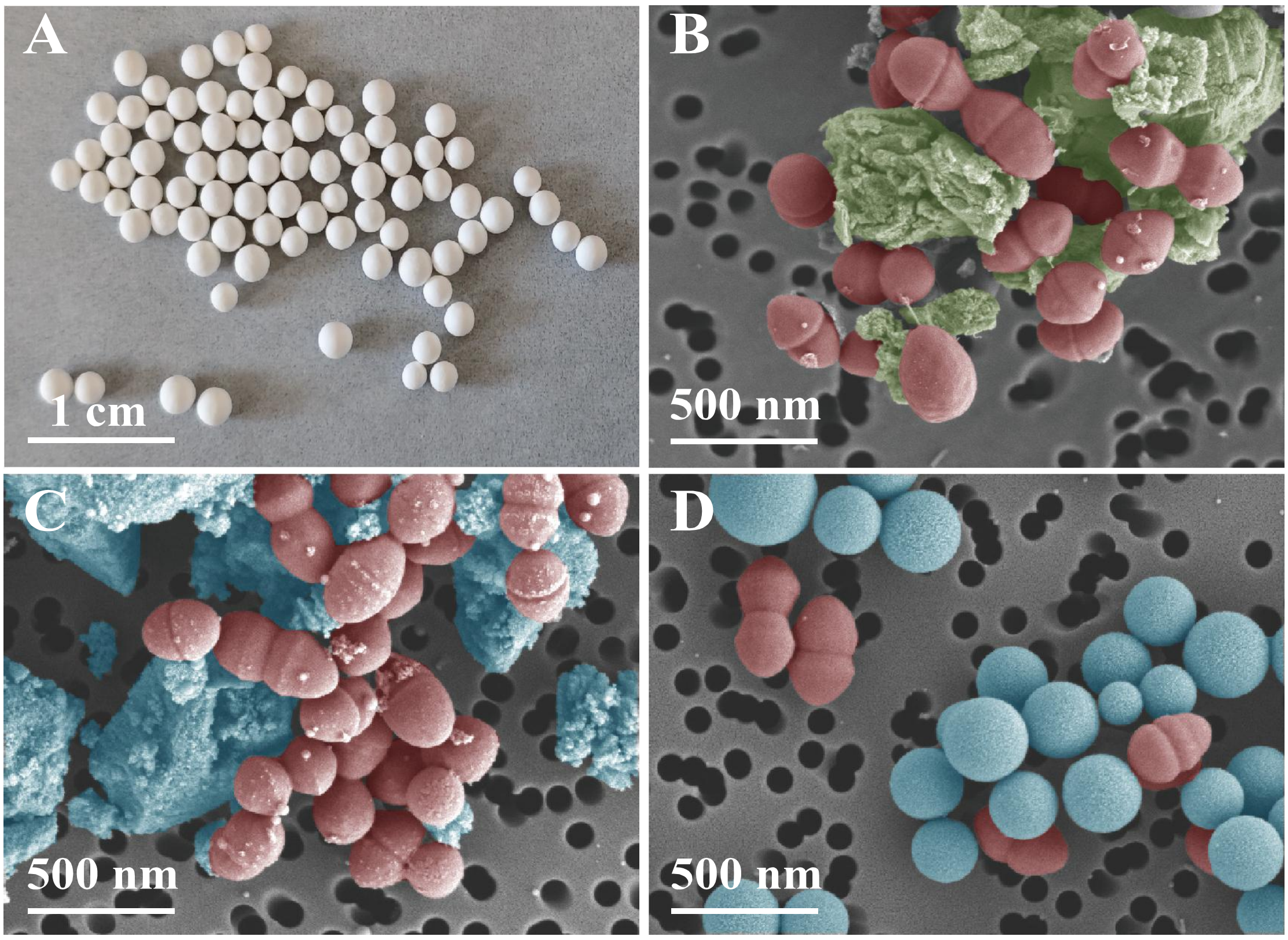

Fig. S1 Photograph of (A) 3 mm Al_2O_3 mineral; and SEM microphotograph showing *E. faecalis* cells treated with (B) 3 mm Al_2O_3, (C) 44 µm SiO_2, and (D) 200 nm SiO_2. Due to the large size on the millimeter scale, the Al_2O_3 particles cannot be fully captured within a single SEM image. Therefore, figure (A) shows a picture of 3 mm Al_2O_3 material. To facilitate visual identification, false color labeling was applied to cells and mineral particles, with *E. faecalis* rendered in red, Al_2O_3 in green, and SiO_2 in blue.

Fig. S2

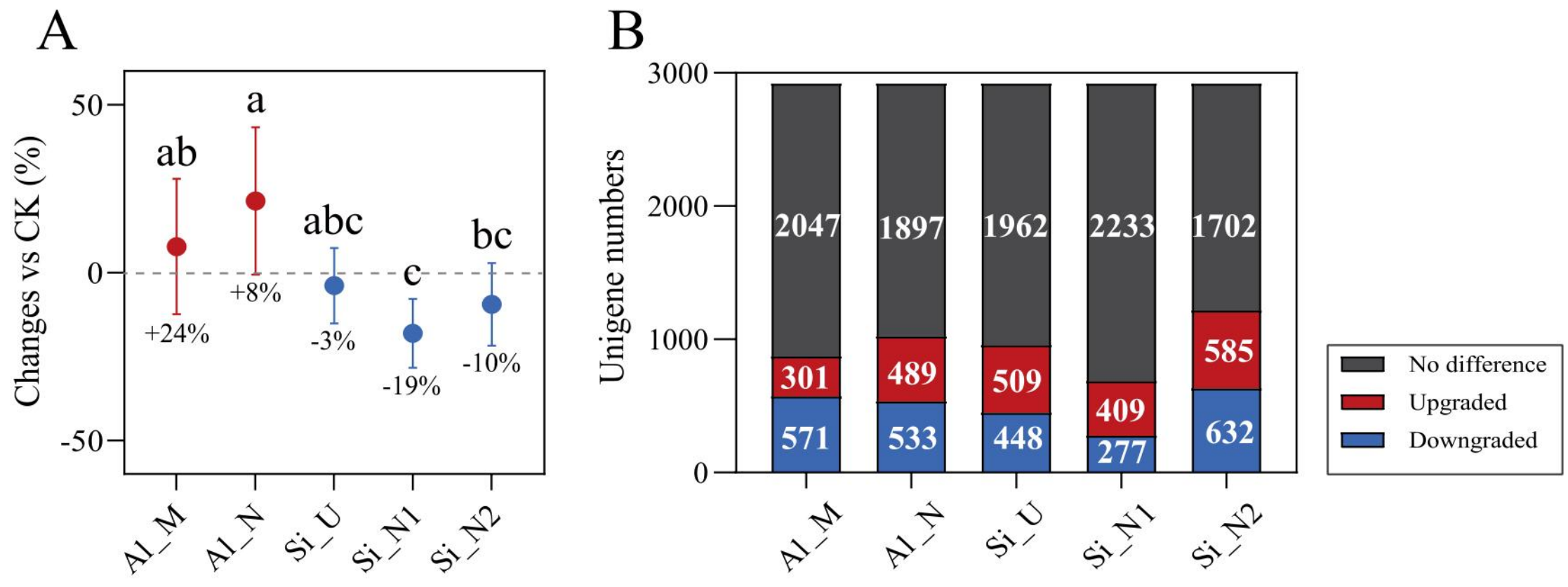

Fig. S2 Effects of Al_2O_3 and SiO_2 on the (A) viability and (B) gene expression of *E. faecalis* during the logarithmic growth phase. Changes in viability are expressed as percentage difference relative to control. Notice the opposite effects of Al_2O_3 and SiO_2, particularly of nanoparticles. Lowercase letters in (A) indicate significant differences among treatments ($P < 0.05$). Upgraded and downgraded genes in (B) are defined as those with $|Log_2FC| > 0.5$ and $FDR < 0.05$.

Fig. S3

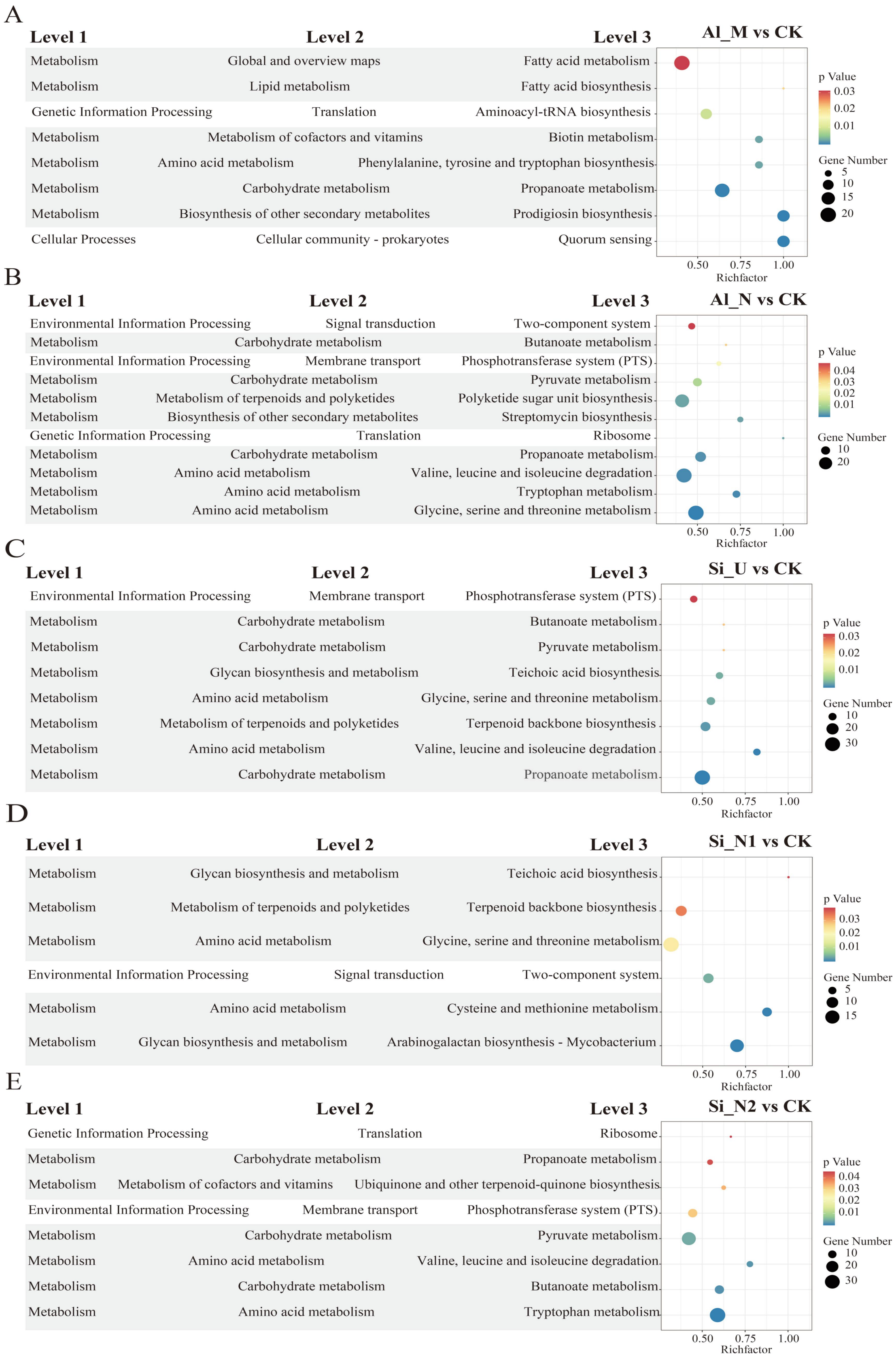

Fig. S3 KEGG pathway enrichment analysis of differentially expressed genes

(DEGs) under (A) Al_M, (B) Al_N, (C) Si-U, (D) Si_N1 and (E) Si_N2 treatment. The dot size represents the number of DEGs, and the dot color indicates the P-value of each pathway calculated based on the hypergeometric distribution. The pathways with P-value < 0.05 were significantly enriched pathways. Rich factor is the ratio of DEGs numbers annotated in this pathway term to all gene numbers in the same pathway term.

Fig. S4

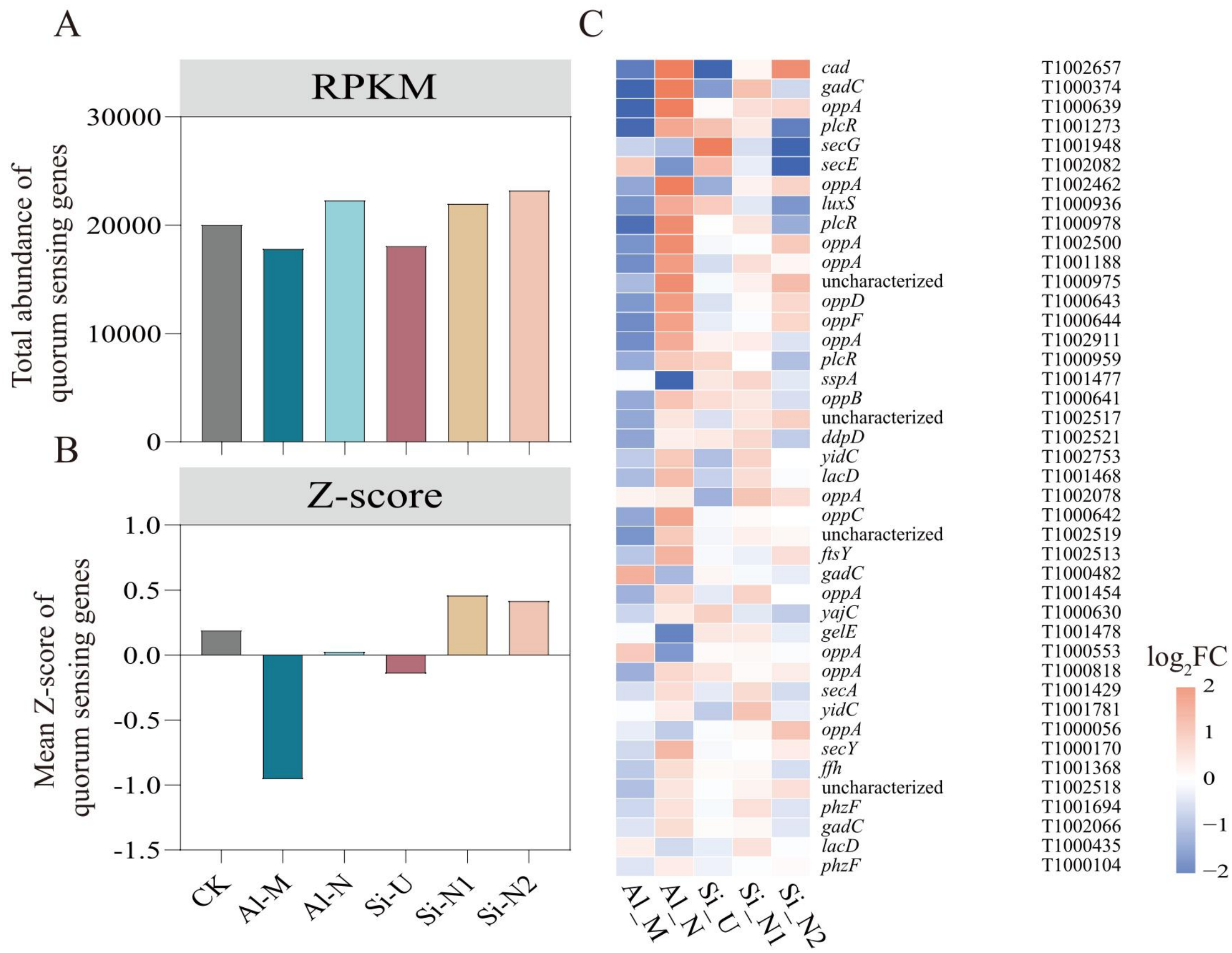

Fig. S4 Effects of Al_2O_3 and SiO_2 on (A) total expression, (B) mean Z-score, and (C) fold changes in gene abundance of quorum sensing-related genes in *E. faecalis*. Data are shown as mean ± standard deviation of three independent biological replicates (n = 3). Color intensity in (C) reflects expression changes according to the scale shown (red, up regulation; blue, down regulation).

Fig. S5

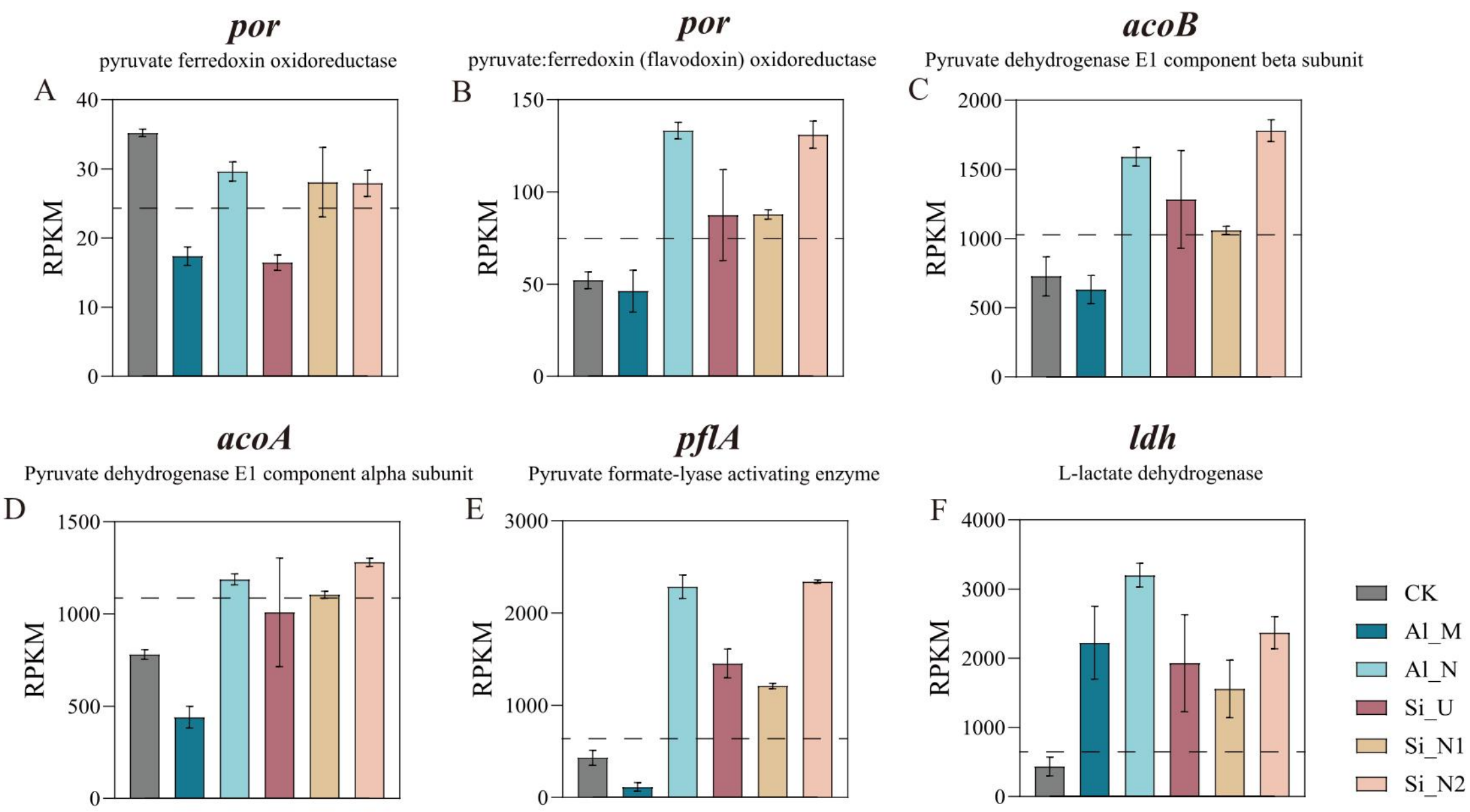

Fig. S5 Effects of Al_2O_3 and SiO_2 on gene transcription relative to (A-E) pyruvate metabolism and (F) lactate conversion. Dotted line represents $|\log_2 FC| = 0.5$ compared with the CK group. Data are shown as mean ± standard deviation of three independent biological replicates (n = 3).

Fig. S6

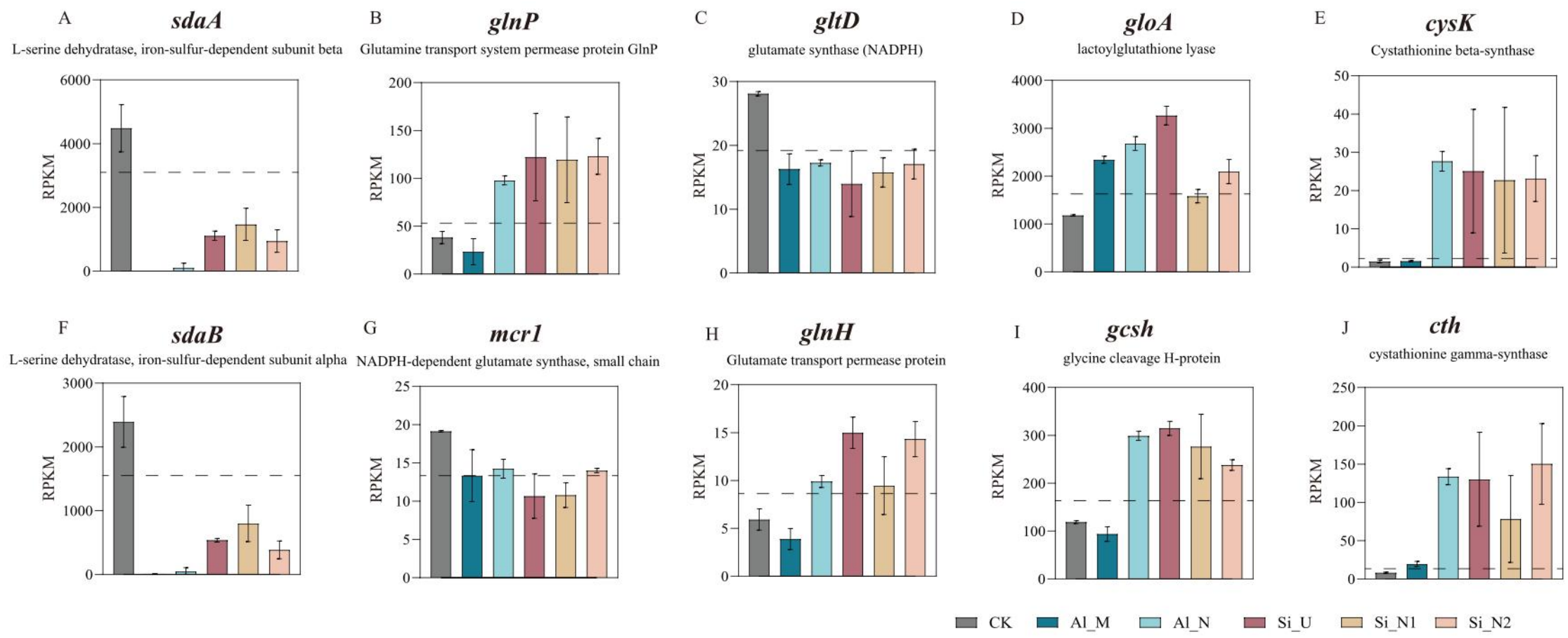

Fig. S6 Effects of Al_2O_3 and SiO_2 on gene transcription relative to (A-J) amino acid metabolism. Dotted line represents $|\log_2 FC| = 0.5$ compared with the CK group. Data are shown as mean ± standard deviation of three independent biological replicates (n = 3).

Fig. S7

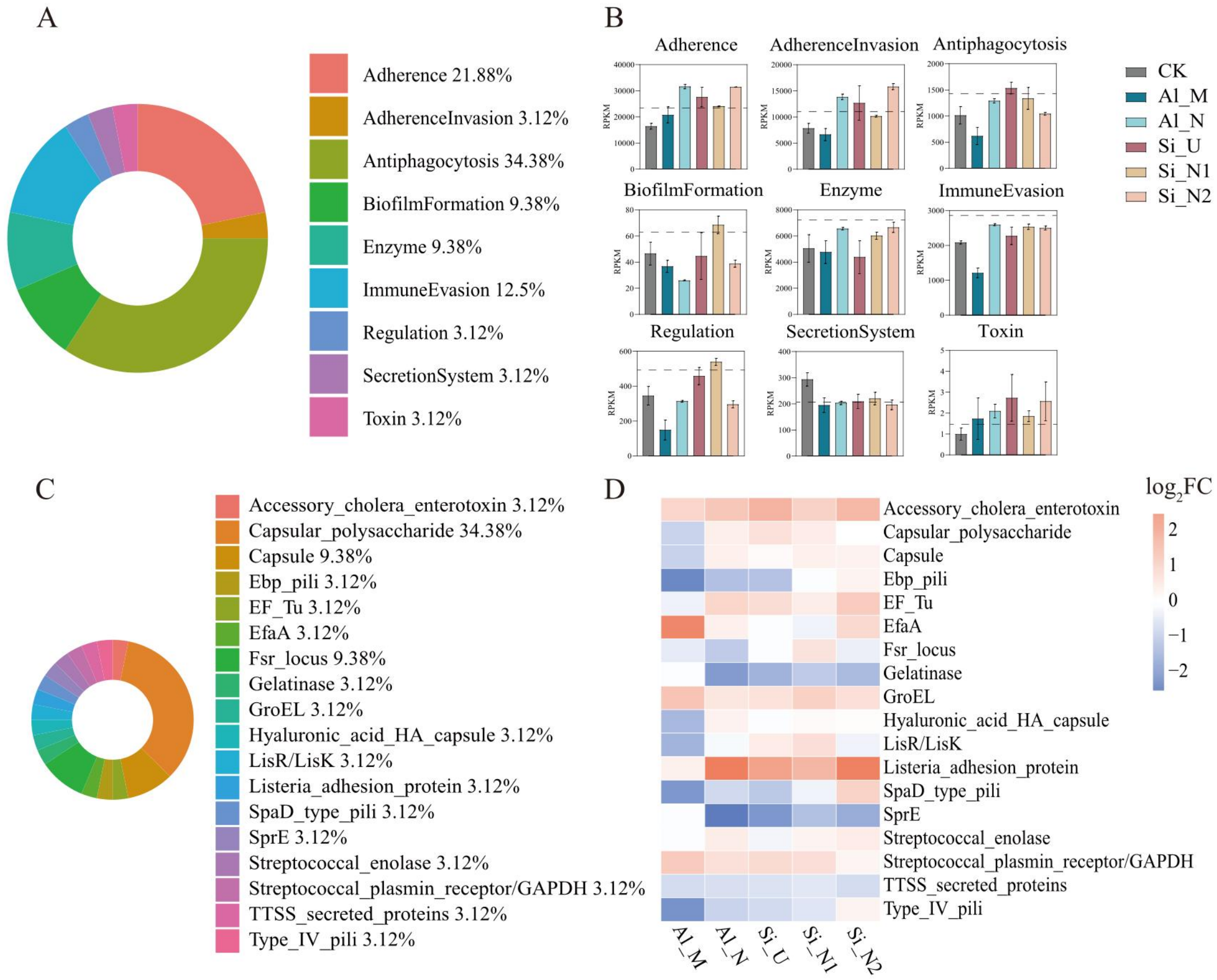

Fig. S7 Effects of Al_2O_3 and SiO_2 on the (A-B) types and (C-D) subtypes of virulence factors in *E. faecalis* as annotated by the VFDB Database. (A) and (C) show the distribution and relative abundance of VFDB-annotated virulence factors, based on the note shown. The dotted line in (B) represents $|log_2FC| = 0.5$ compared with the CK group. Data are shown as mean ± standard deviation of three independent biological replicates (n = 3). Color intensity in (D) reflects expression changes according to the scale shown (red, up regulation; blue, down regulation).

Fig. S8

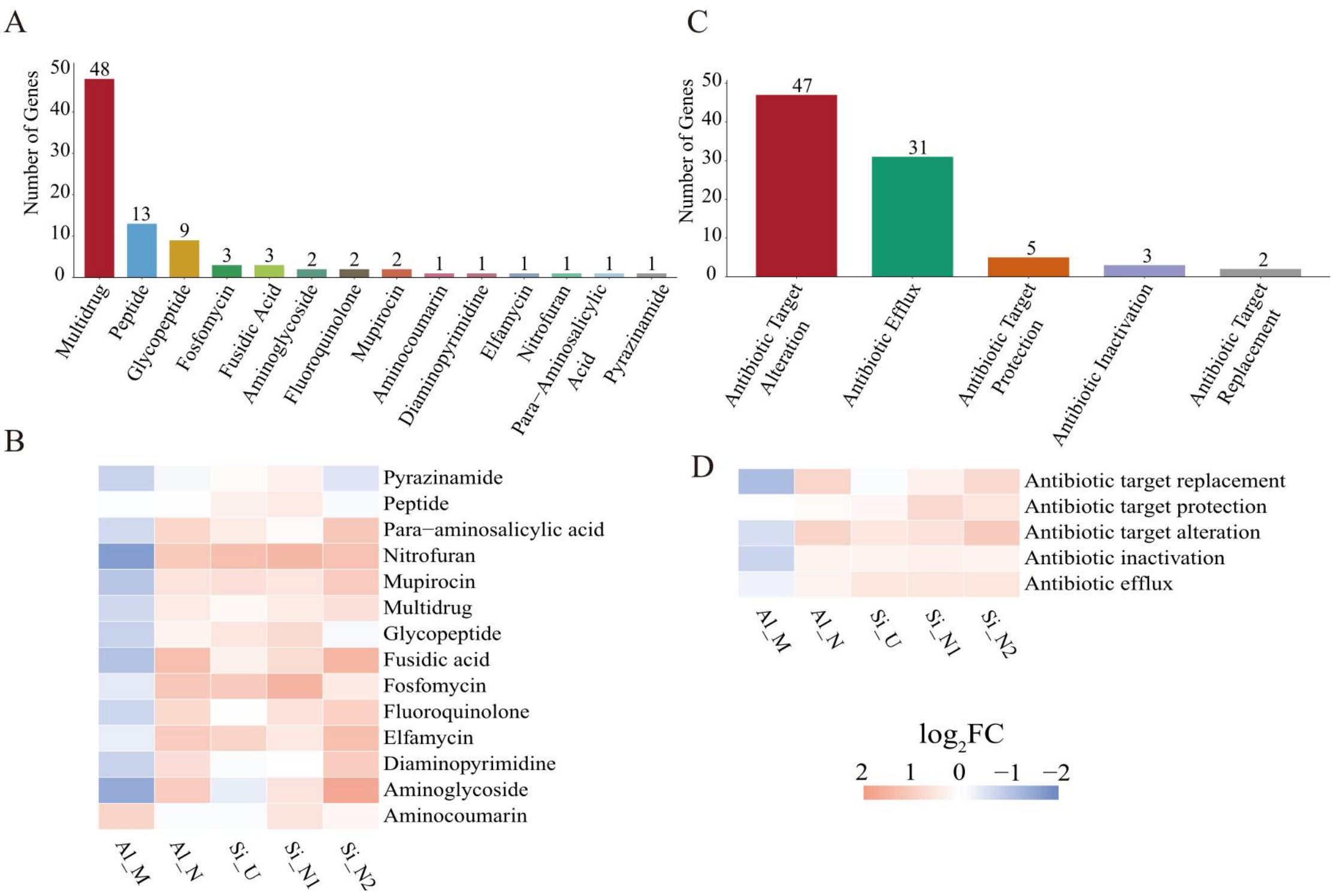

Fig. S8 Effects of Al_2O_3 and SiO_2 on the (A-B) drug classes and (C-D) resistance mechanisms in *E. faecalis* as annotated by the CARD database. Color intensity in (B) and (D) reflects expression changes according to the scale shown (red, up regulation; blue, down regulation). Notably, antibiotic resistance in *E. faecalis* was enhanced by all mineral treatments except for Al_M.
